# Beam Propagation Method Calculating Attenuated Total Reflection Spectra to Excite Hybridized Surface Plasmon Polaritons

**DOI:** 10.3390/ma8085048

**Published:** 2015-08-07

**Authors:** Hongli Zhou, Xueru Zhang, Yuxiao Wang, Yinglin Song

**Affiliations:** 1 Department of Physics, Harbin Institute of Technology, 92 West Dazhi Street, Harbin 150001, China; E-Mails: zhoumiao008@163.com (H.Z.); wangyx@hit.edu.cn (Y.W.); 2 Department of Physics, Anhui University of Science & Technology, 168 Shungeng Road, Huainan 232001, China

**Keywords:** beam propagation method, attenuated total reflection spectrum, hybridized surface plasmon polaritons

## Abstract

Using the beam propagation method, an analytical expression of the reflection spectra of a Kretschmann configuration is derived in order to excite hybridized surface plasmonic polaritons (HSPPs). In this configuration, the cladding is a uniaxial dielectric with the optical axis parallel to the interface. The validity of the analytical expression is confirmed by a finite-difference time-domain algorithm and a reported experimental result, respectively. Based on this expression, the properties and the conditions for excitation of the HSPPs are discussed in detail, with regard to the strongly anisotropic cladding and the weakly anisotropic cladding.

## 1. Introduction

In recent years, surface plasmon polaritons (SPPs) at the interface between an anisotropic dielectric and a metal have received increasing attention [1,2,3,4,5]. If the optical axis of the uniaxial dielectric is parallel to the planar interface but not parallel or perpendicular to the propagation direction of SPPs, strictly speaking, only hybridized surface plasmon polaritons (HSPPs) can exist [6,7]. The HSPPs are a mixture [6] of transverse electric (TE) and transverse magnetic (TM) polarization states on the metal side of the interface, and in general a mixture of ordinary and/or extraordinary waves [8,9] on the dielectric side of the interface [1], which are different from conventional TM SPPs. The excitation of the HSPPs can still resort to the attenuated total reflection (ATR) method in the Kretschmann configuration [10], which is a simple and common way. When total reflection of the incident light takes place, the inhomogeneous wave that runs along the boundary between a dense isotropic dielectric and a metal can excite SPPs or HSPPs at the boundary between the metal and a rare dielectric. The resonance case is detected by a deep minimum in the totally reflected beam since the metal absorbs energy. For the Kretschmann configuration with a uniaxial cladding where the optical axis lies in the plane of the interface, the calculation of the ATR spectrum is considerably more complicated. The calculation of the ATR in the situation can be carried out by the transfer matrix method [11], the exact coupled wave analysis [12], or a finite-difference time-domain (FDTD) algorithm. However, any one of these approaches, although effective, is not a simple analytic theory [7]. Therefore, up to now, there have been no analytical expressions in the literature to compute the reflectance spectra of the Kretschmann configuration with an anisotropic cladding.

In this paper, we propose an alternative approach to calculate the reflectance spectra in the Kretschmann configuration, where the optical axis of the uniaxial dielectric cladding is parallel to the interface. The beam propagation method (BPM) is used to derive an analytical expression of the reflectivity of this configuration. The validity of the expression of the reflectivity is confirmed in two different ways. The BPM can avoid dealing with the complex situation that the electric fields of the refractive rays in anisotropic media do not lie in the plane of incident. Utilizing the ATR spectra, we analyze how the hybridized surface plasmon resonance relates to the degree of anisotropy of a uniaxial dielectric cladding and the azimuth angle of its optical axis.

## 2. Theoretical Section

**Figure 1:**
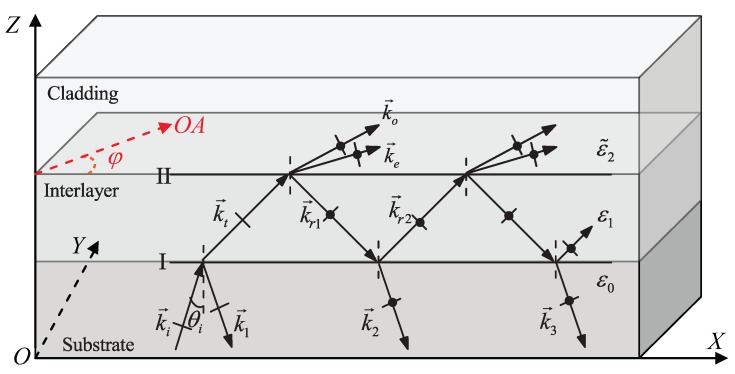
Reflection of a transverse magnetic (TM) plane wave in a three-layer dielectric system, where both the substrate and the interlayer are isotropic media, and the cladding is a uniaxial dielectric with optical axial (OA) parallel to the interface.

In this section, to calculate the ATR spectrum of the Kretschmann configuration to excite HSPPs, we firstly derive the reflectivity of a three-layer dielectric system ($\varepsilon_{0}$/$\varepsilon_{1}$/${\tilde{\varepsilon }}_{2}$) by means of the BPM, whose schematic diagram is shown in Figure 1. Then, we make the expressions of the reflectivity extend to the Kretschmann configuration with a metal interlayer. Finally, the validity of the expressions for the reflectivity is proved by the FDTD method and the reported experimental result.

### 2.1. Reflectivity Expressions of the Kretschmann Configuration

In Figure 1, a uniaxial dielectric cladding and a rare isotropic dielectric film are deposited on a dense isotropic dielectric substrate, where their dielectric constants are ${\tilde{\varepsilon }}_{2}$, $\varepsilon_{1}$ and $\varepsilon_{0}$ from up to down respectively. Their dielectric constants satisfy the relations: $\varepsilon_{1}<\left(\varepsilon_{o},\varepsilon_{e}\right)<\varepsilon_{0}$, where $\varepsilon_{o},\varepsilon_{e}$ are the principal dielectric constants of the uniaxial dielectric cladding. The interface I exists between the substrate and the interlayer. The optical axis of the uniaxial dielectric cladding is parallel to the interface II between the uniaxial cladding and the interlayer dielectric. A monochromatic TM plane wave is incident from the substrate to the interlayer, where the complex amplitude of its electric field is expressed as

(1) $${\tilde{U}}_{0}=A_{∥}\exp\left[i\left({\vec{k}}_{i}\cdot \vec{r}-\omega t\right)\right]$$

where $A_{∥}$ is a complex-constant amplitude and ${\vec{k}}_{i}$ is the incident wave vector propagating at an incident angle $\theta_{i}$.

At interface I, the incident wave is divided into the reflected wave ${\vec{k}}_{1}$ and the refracted wave ${\vec{k}}_{t}$. The wave ${\vec{k}}_{t}$ incident at interface II is partly reflected back at interface I, and the other part gives rise to two refracted waves $\left({\vec{k}}_{o},{\vec{k}}_{e}\right)$ in the cladding. The wave ${\vec{k}}_{r1}$ is divided into the refractive wave ${\vec{k}}_{2}$ and reflected wave ${\vec{k}}_{r2}$. The process of the division of the wave remaining in the interlayer continues as described in Figure 1. It is to be noted that, when a TM wave is incident from the interlayer to the cladding, the reflected fields at interface II are a superposition of TE and TM waves. In other words, in this system with anisotropic cladding, the polarization of reflected waves is different from that of incident waves. Similarly, for TE input light, the same results are expected. For analysis convenience, we divide the superposition fields emitted from the interlayer to the substrate into TM and TE waves.

In our three-layer dielectric system, we mark the amplitude reflection coefficient *r* and the amplitude transmission coefficient *t* as $r_{\mathrm{ij},\mathrm{pq}}$ and $t_{\mathrm{ij},\mathrm{pq}}$, where subscripts i, j = 0,1,2, and the sequence order of i and j means that the former is an incident medium and the latter is a refractive medium. The subscripts p and q denote a TM or TE wave, respectively. The sequence order of p and q denotes that the former is the polarization state of the incident wave and the latter is the polarization state of the wave reflected or refracted. That is, p, q = TM, TE. It is well known that *r* and *t* are given by the Fresnel formulas [13] at the interface between two kinds of isotropic media. While, for *r* and *t* at the interface between an isotropic medium and a uniaxial dielectric, one can refer to [9].

For brevity in the derivation, we define the following coefficients $A_{1}$, $A_{2}$, $a_{1}$, $a_{2}$, $b_{1}$, $b_{2}$, $c_{1}$ and $c_{2}$:

(2) $$A_{1}=A_{∥}t_{01,\mathrm{TMTM}}t_{10,\mathrm{TMTM}}$$

(3) $$A_{2}=A_{∥}t_{01,\mathrm{TMTM}}t_{10,\mathrm{TETE}}$$

(4) $$a_{1}=r_{12,\mathrm{TMTM}}^{2}r_{10,\mathrm{TMTM}}-r_{12,\mathrm{TMTE}}^{2}r_{10,\mathrm{TETE}}$$

(5) $$a_{2}=\left(r_{12,\mathrm{TMTM}}r_{10,\mathrm{TMTM}}+r_{12,\mathrm{TETE}}r_{10,\mathrm{TETE}}\right)r_{12,\mathrm{TMTE}}$$

(6) $$b_{1}=r_{10,\mathrm{TMTM}}r_{12,\mathrm{TMTM}}$$

(7) $$b_{2}=r_{10,\mathrm{TETE}}r_{12,\mathrm{TETE}}$$

(8) $$c_{1}=r_{10,\mathrm{TMTM}}r_{12,\mathrm{TMTE}}$$

(9) $$c_{2}=r_{10,\mathrm{TETE}}r_{12,\mathrm{TMTE}}$$

When a TM plane wave is incident from the substrate to the interplayer, the complex amplitudes of the TM waves reflected from the interlayer include the following terms: $A_{∥}r_{01,\mathrm{TMTM}}$, $A_{1}r_{12,\mathrm{TMTM}}e^{i\delta }$, $A_{1}a_{1}e^{i2\delta }$, $A_{1}\left(a_{1}r_{10,\mathrm{TMTM}}r_{12,\mathrm{TMTM}}+a_{2}r_{10,\mathrm{TETE}}r_{12,\mathrm{TETM}}\right)e^{i3\delta }$, $A_{1}[\left(a_{1}r_{10,\mathrm{TMTM}}r_{12,\mathrm{TMTM}}+a_{2}r_{10,\mathrm{TETE}}r_{12,\mathrm{TETM}}\right)r_{10,\mathrm{TMTM}}r_{12,\mathrm{TMTM}}+(a_{1}r_{10,\mathrm{TMTM}}r_{12,\mathrm{TMTE}}+a_{2}r_{10,\mathrm{TETE}}r_{12,\mathrm{TETE}})r_{10,\mathrm{TETE}}r_{12,\mathrm{TETM}}]e^{i4\delta }$, etc. Similarly, in the substrate, the complex amplitudes of the TE waves reflected from the interlayer are expressed as $A_{2}r_{12,\mathrm{TMTE}}e^{i\delta }$, $A_{2}a_{2}e^{i2\delta }$, $A_{2}\left(a_{1}r_{10,\mathrm{TMTM}}r_{12,\mathrm{TMTE}}+a_{2}r_{10,\mathrm{TETE}}r_{12,\mathrm{TETE}}\right)e^{i3\delta }$, $A_{2}\left[\left(a_{1}r_{10,\mathrm{TMTM}}r_{12,\mathrm{TMTM}}+a_{2}r_{10,\mathrm{TETE}}r_{12,\mathrm{TETM}}\right)r_{10,\mathrm{TMTM}}r_{12,\mathrm{TMTE}}+\left(a_{1}r_{10,\mathrm{TMTM}}r_{12,\mathrm{TMTE}}+a_{2}r_{10,\mathrm{TETE}}r_{12,\mathrm{TETE}}\right)r_{10,\mathrm{TETE}}r_{12,\mathrm{TETE}}\right]e^{i4\delta }$, etc. The *δ* is the phase difference between adjacent beams in the substrate

(10) $$\delta =2k_{i}d{\left(\varepsilon_{1}/\varepsilon_{0}-\sin^{2}\theta_{i}\right)}^{1/2}$$

where *d* is the thickness of the interlayer.

If all TM and TE reflected waves in the substrate are superimposed separately, the total complex amplitudes of TM and TE waves of the three-layer dielectric system can be expressed as

(11) $${\tilde{U}}_{R,\mathrm{TM}}=A_{∥}r_{01,\mathrm{TMTM}}+A_{1}e^{i\delta }\left[r_{12,\mathrm{TMTM}}+a_{1}e^{i\delta }+\frac{\left(a_{1}b_{1}-a_{2}c_{2}\right)e^{i2\delta }}{\left(1-b_{1}e^{i\delta }\right)\left(1+c_{1}c_{2}e^{i2\delta }\right)}-\frac{\left(a_{2}b_{2}+a_{1}c_{1}\right)c_{2}e^{i3\delta }}{\left(1-b_{1}e^{i\delta }\right)\left(1-b_{2}e^{i\delta }\right)}\right]$$

(12) $${\tilde{U}}_{R,\mathrm{TE}}=A_{2}e^{i\delta }\left[r_{12,\mathrm{TMTE}}+a_{2}e^{i\delta }+\frac{\left(a_{1}c_{1}+a_{2}b_{2}\right)e^{i2\delta }}{\left(1-b_{2}e^{i\delta }\right)\left(1+c_{1}c_{2}e^{i2\delta }\right)}+\frac{\left(a_{1}b_{1}+a_{2}c_{2}\right)c_{1}e^{i3\delta }}{\left(1-b_{1}e^{i\delta }\right)\left(1-b_{2}e^{i\delta }\right)}\right]$$

Therefore, the total reflectance in the three-layer dielectric system is given by the sum of TE and TM reflectance

(13) $$R_{\mathrm{total}}\left(\theta_{i},\varphi \right)=R_{\mathrm{TM}}+R_{\mathrm{TE}}$$

(14) $$R_{\mathrm{TM}}={\left|\frac{{\tilde{U}}_{R,\mathrm{TM}}}{A_{∥}}\right|}^{2}$$

(15) $$R_{\mathrm{TE}}={\left|\frac{{\tilde{U}}_{R,\mathrm{TE}}}{A_{∥}}\right|}^{2}$$

where *φ* is the azimuth angle between the direction of the optical axis in the interface and the plane of incident; and $R_{\mathrm{TM}}$ and $R_{\mathrm{TE}}$ represent the reflectance of TM and TE waves in the substrate for a TM incident wave, respectively. It is evident, when the thickness of the interlayer *d* is of known, that the total reflectivity $R_{\mathrm{total}}\left(\theta_{i},\varphi \right)$ is a function of the incident angle $\theta_{i}$ and the azimuth angle of the optical axis *φ* in this system.

As is well known, the theory of the multiple-beam interference with a plane-parallel plate keeps its validity for a three-layer system composed of a dense isotropic dielectric substrate, a plane-parallel metal film and a uniaxial dielectric cladding with the optical axis parallel to the interface, if it only involves linear relations between field vectors of a time-harmonic wave [13]. Therefore, if the real dielectric constant $\varepsilon_{1}$ in expressions (2)–(12) is replaced by a complex dielectric constant of a metal film, we can get the reflectivity of the Kretschmann configuration with an anisotropic cladding in the case of a TM plane wave incident from the substrate.

### 2.2. Verification of the Reflectivity Expressions

In order to evaluate the accuracy of the analytical method, several key comparisons between two ATR curves based on the BPM and the FDTD method, and based on the BPM and an experiment reported in the paper [2] are implemented. We firstly calculate the ATR spectrum of the Kretschmann configuration by the reflectivity expression (13). Then the numerical simulation by means of the FDTD method is performed by the commercial software FDTD Solution (FDTDS). A TM plane wave incident from the substrate is kept in the simulation and the parameters of materials chosen in the simulations are separated into two sets. The parameters of one set with the strongly anisotropic cladding are as follows [7] :

(1) The substrate dielectric constant is $\varepsilon_{0}$ = 12;
(2) The dielectric constant of Au at the wavelength 1.0 *μ*m is $\varepsilon_{1}=-42+2.9$i, and the thickness of the Au film is *d* = 50 nm;
(3) The principal dielectric constants of the uniaxial dielectric are $\varepsilon_{o}=2.0$ and $\varepsilon_{e}=7.0$;
(4) Considering the conditions for excitation of HSPPs, choose the azimuth angle of the optical axis $\varphi =30^{\circ }$, $50^{\circ }$, $70^{\circ }$, respectively, and change the incident angle $\theta_{i}$ from 0 to 90${}^{\circ }$.

The parameters of the other set with the weakly anisotropic cladding are follows [2]:

(1) The dielectric constant of ZF7 as the substrate is $\varepsilon_{0}=1.798^{2}$ at $\lambda_{0}$ = 650 nm.
(2) The dielectric constants of silver at the wavelength from 620 to 730 nm are from the experimental data [14], and the thickness of the Ag film is *d* = 57 nm.
(3) The azobenzene polymer is used as the cladding. When a pump laser irradiates the cladding, its principal dielectric constants are $\varepsilon_{o}=1.5228^{2}$ and $\varepsilon_{e}=1.5124^{2}$. While, the dielectric constant without the pump light is $\varepsilon_{2}=1.5262^{2}$, standing for an isotropic medium.
(4) As the same as the conditions of the paper [2], we also set the condition that no pump, φ=0, $60^{\circ }$, and $90^{\circ }$, respectively, and keep the incident angle $\theta_{i}$ at a fixed value of $63.89^{\circ }$.

**Figure 2:**
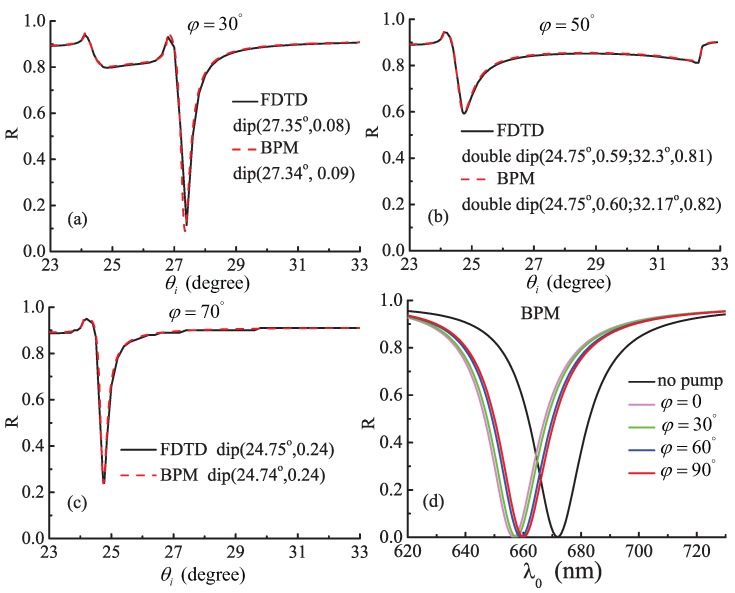
Comparison between attenuated total reflection (ATR) curves from the beam propagation method (BPM) and the standard which are the finite-difference time-domain (FDTD) method and the results of the experiment in the paper [2], respectively. The first three graphs compare with the result of FDTD method; while the last one may be compared with the result of the experiment in the paper [2]. The total reflectivity *R_total_* of this system is a function of the azimuth angle of the optical axis *φ*, incident angles $\theta_{i}$ and the resonance wavelength $\lambda_{0}$.

**Figure 3:**
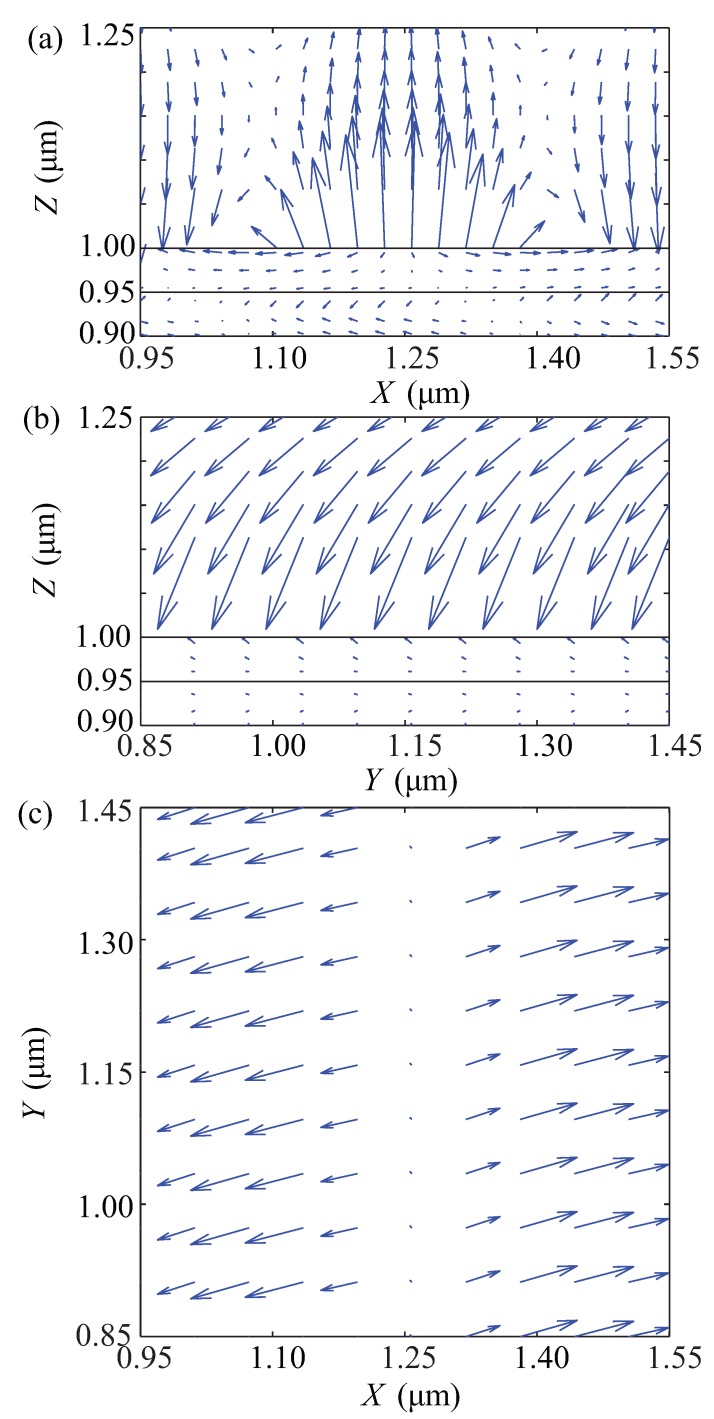
Field vector plots of the electric field *E* for hybridized surface plasmonic polaritons (HSPPs) excited at the reflectance dip in Figure 2a. (**a**) $\vec{E}\times 3.5$ in XZ plane; (**b**) $\vec{E}\times 3.5$ in YZ plane; (**c**) in XY plane. Values of the electric field in XZ plane and YZ plane are expanded by factors of 3.5 for visual purposes. The electric field vectors are scaled arbitrarily and arrows show their direction.

The total reflectivity $R_{\mathrm{total}}\left(\theta_{i},\varphi \right)$ as a function of the incident angles $\theta_{i}$ and the azimuth angle of the optical axis *φ* is displayed in Figure 2, when a TM light is incident this system. In the first three graphs Figure 2a–c corresponding to the parameters of the first set materials, the black solid and the red dash lines are the results of the FDTDS and the expression (13) derived by the BPM, respectively. According to the resonance angle, the value of $R_{\mathrm{total}}$ in the dip of the reflectivity, and the shape of curves, it is evident that the results of the BPM excellently agree with the ones of the FDTD. The parameters of the last graph Figure 2d are in accordance with those of the second set materials, and the color curves are calculated by the expression (13). Under different conditions with no pump, φ=0, $30^{\circ }$, $60^{\circ }$, and $90^{\circ }$, the resonance wavelengths are 672, 656, 658, 659, 660 nm, respectively. Compared with the Figure 3a in the paper [2], the change tendency of the resonance wavelengths is quite consistent, although the different values are approximately equal to 10 nm. The results indicate that the expressions (11)–(13) can accurately compute the ATR curves of this system for any parameters. Furthermore, using the analytical expressions it takes only several seconds to describe one ATR curve on a PC equipped with 8 Inter(R) Core i7-2600 CPUs @ 3.4 GHz and 16 GB RAM.

## 3. Results and Discussion

### 3.1. Field Distribution and Polarization of the HSPPs

**Figure 4:**
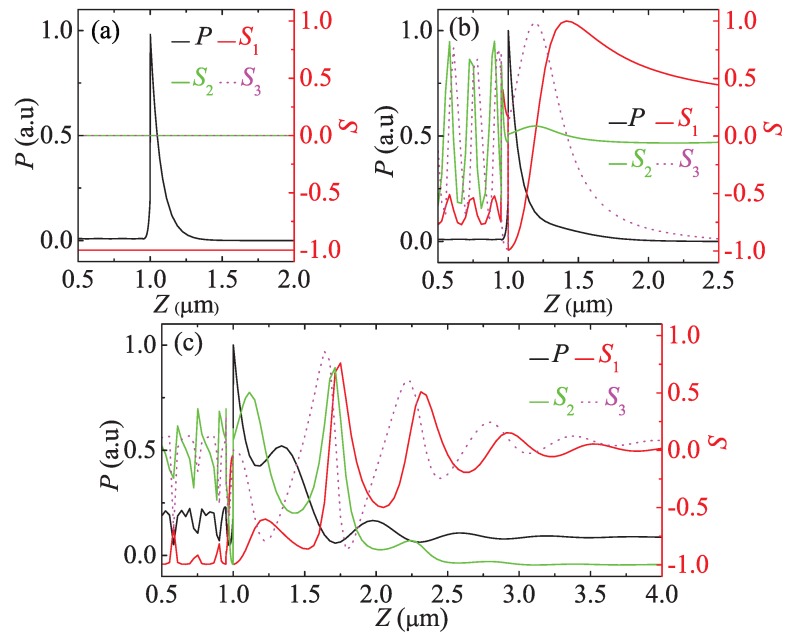
Lateral distribution of Poynting vectors (*P*) and Stocks parameters (S1,S2, and S3) for the SPPs and the HSPPs excited at the Kretschmann configuration. (**a**) φ=0, TM SPPs; (**b**) $\varphi =30^{\circ }$, the HSPPs excited at the reflectance dip in Figure 2a; (**c**) $\varphi =50^{\circ }$, the HSPPs excited at the left reflectance dip in Figure 2b.

To investigate the properties of the HSPPs, we consider the HSPPs excited at the reflectance dips in Figure 2a,b (the left one) for the Kretschmann configuration. The region of the metal film in the interlayer is from 0.95 *μ*m to 1.0 *μ*m along the *Z* axis. The parameters of the materials used in the configuration are kept as the first set above. By means of extracting the components of the electric field through the point (1.0, 1.0, 1.0) *μ*m in the coordinate system, the field distributions, polarization and the Poynting vector of the HSPPs are numerically simulated by the FDTDS, as shown in Figure 3 and Figure 4, respectively. In addition, to compare the properties of the HSPPs and the TM SPPs, the TM SPPs is excited when φ=0 and $\theta_{i}=26.35^{\circ }$ in the same configuration and the parameters of the materials above. To sum up, we choose three kinds of conditions for exciting TM SPPs or HSPPs.

The field vector plots of the electric field of the HSPPs excited at the dip in Figure 2a are illustrated in Figure 3, which possess an obvious characteristic. It is clear that the components of the electric fields are non-zero in the three directions. The $E_{y}$ relative to the $E_{x}$ and the $E_{z}$ is not negligible. The electric field strength *E* decreases exponentially with the distance *Z* from the surface in Figure 3a,b, which is similar to that of the TM SPPs.

For the polarization of the HSPPs, we individually simulate the Stocks parameters and the lateral distributions of Poynting vectors of the HSPPs and the TM SPPs excited on three conditions above. According to the Stocks parameters describing the polarization state of the optical field [15], we find from Figure 4a that the TM SPPs show the vertically linearly polarized light and strong localization. However, the HSPPs in Figure 4b demonstrate elliptical polarization state and good localization, which displays a nonradiative characteristic. And the HSPPs in Figure 4c gradually change from elliptical polarization into -45${}^{\circ }$ linear polarization along +*Z* in the uniaxial cladding. It is noted that the Poynting vector oscillates with an exponentially decaying in Figure 4c, and it is nonzero up to the 4 *μ*m. This means that the leakage of energy is along the +*Z* in the cladding.

### 3.2. HSPPs Excited in the Kretschmann with Strongly Anisotropic Dielectric Claddings

On the basis of the different properties of the HSPPs above, we will identify the conditions for the excitation of the HSPPs with different properties in the Kretschmann configuration with a strongly anisotropic dielectric cladding.

**Figure 5:**
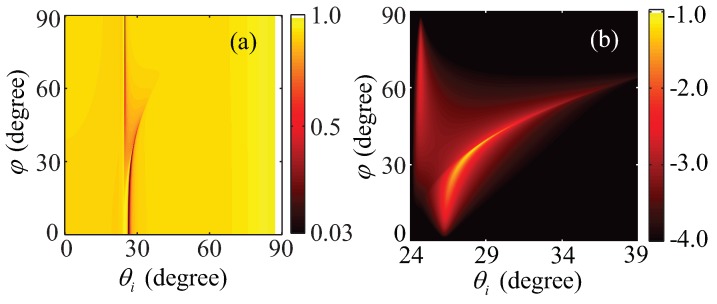
Reflectance spectra of the Kretschmann configuration with a strongly electrically anisotropic uniaxial dielectric cladding. (**a**) The total reflectivity $R_{\mathrm{total}}\left(\theta_{i},\varphi \right)$ as a function of the incident angle ($\theta_{i}$) and the azimuth angle *φ* between the optical axis and the +*X* axis; (**b**) Detail of the part $R_{\mathrm{TE}}$ in (**a**), and the color bar on a log 10 scale, *i.e.*, lg($R_{\mathrm{TE}}$).

Utilizing reflectivity expressions (13) and (15), the ATR spectra are demonstrated in Figure 5, where the parameters of the materials are kept as the first set mentioned above. The trace of low values of the reflectivity denotes the positions where the surface plasmon resonance takes place. To be specific, every azimuth angle *φ* between 30${}^{\circ }$ and 60${}^{\circ }$ does correspond to two resonance angles. The split of the trace results from the difference between critical angles of total reflection of the ordinary wave and the extraordinary wave, where the critical angle of total reflection of the latter varies with the rotation of the optical axis.

In Figure 5a, the right trace of the low values of the reflectivity corresponds to the condition for the excitation of the HSPPs with the properties shown in Figure 4b. The HSPPs is excited by coupling of the evanescent waves of both extraordinary wave and ordinary wave. For the left trace of the low values of the reflectivity, we separate it into two regions to analyze the condition for excitation of the HSPPs. When the azimuth angle is between 30${}^{\circ }$ and 60${}^{\circ }$, the HSPPs is excited by coupling the evanescent waves of the ordinary and the propagating mode of the extraordinary wave. Thus, the HSPPs have the quasiguided characteristic, and its polarization and localization is similar to Figure 4c. When the azimuth angle is between 60${}^{\circ }$ and 90${}^{\circ }$, the HSPPs is excited mainly by the evanescent waves of the ordinary, where the split of the trace of the low values of the reflectivity gradually degenerates. The properties of the HSPPs are similar to Figure 4b. In addition, Figure 5b indicates the reflectivity spectra $R_{\mathrm{TE}}$. It further shows Figure 5a demonstrates the conditions for excitation of SPPs with the pronounced hybridized characteristic.

From the above discussion, the accuracy conditions for excitation of HSPPs can be directly identified in Figure 5a, including the incident angle and the azimuth angle. Associating Figure 5a,b, one can know properties of the HSPPs and the corresponding conditions for excitation. However, using the total reflectance in the Kretschmann configuration as a function of wave vectors in the plane of the interface in [7], it does not directly show the conditions for excitation of HSPPs.

### 3.3. HSPPs Excited in the Kretschmann with Weakly Anisotropic Dielectric Claddings

**Figure 6:**
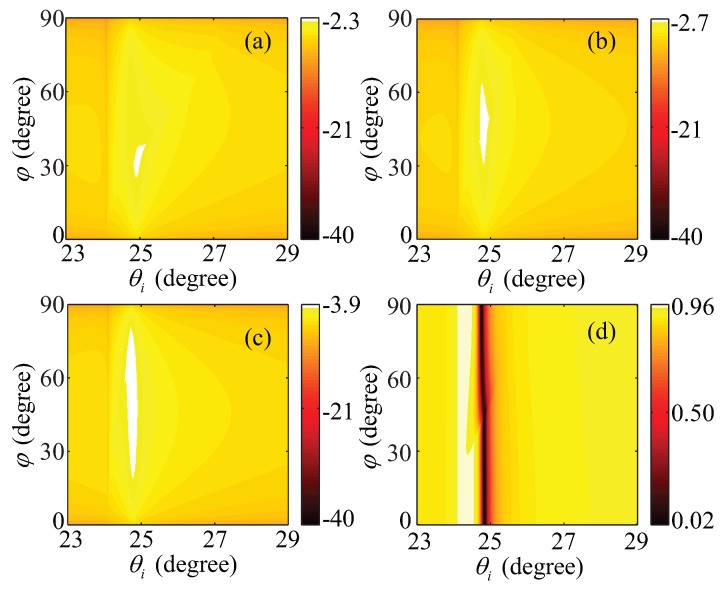
Reflectivity in the Kretschmann configuration with different weakly positive anisotropic uniaxial dielectric claddings. (**a**–**c**) the color bar on a log 10 scale, *i.e.*, lg(*R_TE_*); and (**d**) $R_{\mathrm{TM}}$. And the principal dielectric constants of the claddings are that (**a**) *ε*_o_ = 2.0, *ε*_e_ = 2.5; (**b**,**d**) *ε*_o_ = 2.0, *ε*_e_ = 2.25; and (**c**) *ε*_o_ = 2.0, *ε*_e_ = 2.1, respectively. Other parameters are kept as in Figure 5.

For the case of a natural optical crystal and a weakly anisotropic dielectric, their anisotropy usually does not exceed a few percent. When a TM wave is incident from the isotropic substrate in the Kretschmann configuration with a positive uniaxial weakly anisotropic dielectric cladding, the reflectivity $R_{\mathrm{TE}}$ varying with the degree of anisotropy $|\varepsilon_{o}-\varepsilon_{e}|$ are illustrated in Figure 6. According to the scales of the color bars in Figure 6, we can see when the degree of anisotropy decreases from 0.5 to 0.1, the maximums of $R_{\mathrm{TE}}$ decline from 0.005 to $10^{-4}$, which can be negligible relative to $R_{\mathrm{TM}}$ in Figure 6d. When the azimuth angle of the optical axis changes, the positions of surface plasmon resonance in the ATR spectra approximate a fixed incident angle, which is similar to the case of the isotropic dielectric cladding. These results indicate, at the interface between a metal and a positive uniaxial cladding with weak anisotropy, the HSPPs can be regarded as TM SPPs. In the case of a negative uniaxial cladding with weak anisotropy, one can come to the same result. This approximation is applied in the HSPPs at the interface between a weakly anisotropic dielectric and a metal, so that a simple dispersion relation and equivalent experimental results can be achieved, see, e.g., [2].

## 4. Conclusions

We have proposed an effective calculation and analysis of the ATR spectra in the Kretschmann configuration, where the optical axis of the uniaxial dielectric cladding is parallel to the interface. The analytical expressions for the ATR spectra have been derived based on the BPM, and the validity of the expressions has been confirmed by the FDTD method and the reported experimental results. Utilizing the fast, simple yet reliable computation approach, we demonstrated properties of the HSPPs and analyzed the reflectance spectra of TM and TE waves. The results can directly show the conditions for excitation of the HSPPs. For a positive uniaxial strongly anisotropic dielectric cladding, noradiative HSPPs and quaiguided HSPPs at the interface can be excited. For a uniaxial weakly anisotropic dielectric cladding, the HSPPs can be approximated as TM SPPs. The results can be helpful for designing new materials and structures with HSPPs.
